# Markers of Epstein-Barr Virus Infection in Patients with Multiple Sclerosis

**DOI:** 10.3390/microorganisms11051262

**Published:** 2023-05-11

**Authors:** Cyril Debuysschere, Magloire Pandoua Nekoua, Didier Hober

**Affiliations:** Laboratoire de Virologie ULR3610, Université de Lille, CHU Lille, 59000 Lille, France; cyril_debuysschere@hotmail.com (C.D.); magloire-pandoua.nekoua@univ-lille.fr (M.P.N.)

**Keywords:** Epstein-Barr virus, infection, autoimmunity, multiple sclerosis

## Abstract

Viral infections have been suspected of being involved in the pathogenesis of certain autoimmune diseases for many years. Epstein-Barr virus (EBV), a DNA virus belonging to the *Herpesviridae* family, is thought to be associated with the onset and/or the progression of multiple sclerosis (MS), systemic lupus erythematosus, rheumatoid arthritis, Sjögren’s syndrome and type 1 diabetes. The lifecycle of EBV consists of lytic cycles and latency programmes (0, I, II and III) occurring in infected B-cells. During this lifecycle, viral proteins and miRNAs are produced. This review provides an overview of the detection of EBV infection, focusing on markers of latency and lytic phases in MS. In MS patients, the presence of latency proteins and antibodies has been associated with lesions and dysfunctions of the central nervous system (CNS). In addition, miRNAs, expressed during lytic and latency phases, may be detected in the CNS of MS patients. Lytic reactivations of EBV can occur in the CNS of patients as well, with the presence of lytic proteins and T-cells reacting to this protein in the CNS of MS patients. In conclusion, markers of EBV infection can be found in MS patients, which argues in favour of a relationship between EBV and MS.

## 1. Introduction

**Figure 1:**
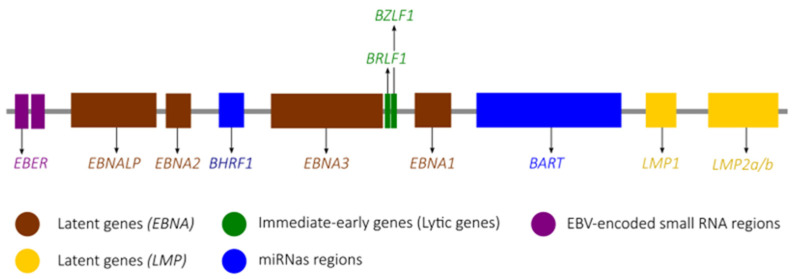
Schematic representation of selected regions of the EBV genome. The six latent genes (*EBNALP*, *EBNA2*, *EBNA3*, *EBNA1*, *LMP1* and *LMP2*) are represented. Immediate-early lytic genes are represented, encoding the transactivators BZLF1 or Zebra protein and BRLF1 or Rta protein, which induce a switch to the lytic cycle. miRNAs coding regions (BART and BHRF1) are also represented. EBNA: Epstein-Barr virus nuclear antigen; LMP: Latent membrane protein; BART: Bam-HI A rightward transcripts; BHFR1: miR-BamHI fragment H rightward open reading frame 1; EBER: EBV-encoded small RNA.

Epstein-Barr virus (EBV), also known as human herpesvirus 4 or lymphocryptovirus human gamma 4, is a linear double-stranded DNA virus that belongs to the *Lymphocrytovirus* genus of the *Gammaherpesvirinae* subfamily within the *Orthoherpesviridae* family [1]. The genome of EBV (~173 kb) encodes approximately 100 proteins and numerous non-coding RNAs and microRNAs (Figure 1) [2,3].

Discovered in 1964 from Burkitt’s lymphoma cell lines [4], this virus has many surprising characteristics: first, it is able, like other viruses of this family, to induce a lifelong latent infection in the host [5]. Secondly, EBV, like Kaposi’s sarcoma-associated herpesvirus and measles virus, is able to infect B-cells, which are the main actors of the humoral adaptive immune response in humans [6,7]. This virus is ubiquitous worldwide, with about 95% of the world’s adult population showing a serological response to EBV [2,8]. It is the causative agent of many diseases. Primo infection, occurring mainly in young children, is often subclinical. In young adults, it mainly presents as infectious mononucleosis with fever, pharyngitis, adenopathy, hepatosplenomegaly and increased fatigue. It is also involved in many cancers, such as Burkitt’s lymphoma, nasopharyngeal carcinoma, Hodgkin’s disease and some gastric cancers [2]. Finally, the primary infection or reactivations may also have a particular presentation in the immunocompromised patient as oral hairy leukoplakia, AIDS-associated B cell lymphoma, post-transplant lymphoproliferative disorder, lymphoid granulomatosis, X-linked lymphoproliferative disorder-associated B-cell and others [9].

For several years, EBV has been suspected to be involved in many autoimmune diseases such as systemic lupus erythematosus (SLE) [10], rheumatoid arthritis (RA) [11], Sjögren’s syndrome [12] and type 1 diabetes [13]. Furthermore, EBV infection has been strongly associated with an increased risk of developing multiple sclerosis (MS) [3,14,15,16,17]. MS is an inflammatory and autoimmune neurological disease, often occurring in young adults, characterised by damages to the central nervous system (CNS) (brain, spinal cord and optic nerves), visible on magnetic resonance imaging (MRI). These lesions are caused by the destruction of the myelin sheath, leading to degeneration of the nerve cells. This disease can take several forms: a relapsing-remitting form (RRMS), the most common one, characterised by periods of disability followed by periods of remission, a progressive form (PPMS), rarer but also worse, where the disability progresses steadily without periods of remission. Finally, a secondary progressive form (SPMS) is characterised by a progressive disability after a few years of remittent phases, where the disability becomes permanent and progressive. The disease may initially present as a clinically isolated syndrome (CIS), a pre-MS syndrome characterised by one or a few isolated neurological symptoms [18].

A longitudinal study including more than 10 million young adults strongly supports the involvement of EBV infection in the pathogenesis of MS. Indeed, EBV seroconversion precedes the onset of MS and is associated with a 32-fold increased risk of developing the disease. Importantly, this risk was not associated with other viruses in this study [8].

Given the recent advances reinforcing the link between EBV infection and MS, this review focuses on markers of EBV infection in patients with MS.

## 2. Life Cycle of EBV

EBV is a strictly human virus mainly transmitted through the saliva of infected persons. The salivary EBV viral load after primary infection can be very high and persist for several months [19]. EBV has a particular tropism for epithelial cells of the oropharynx, as well as for B-cells expressing CD21, which serves as the receptor for virus binding, and MHC class II molecules acting as a co-receptor for the fusion of the virus with the cell [20].

**Figure 2:**
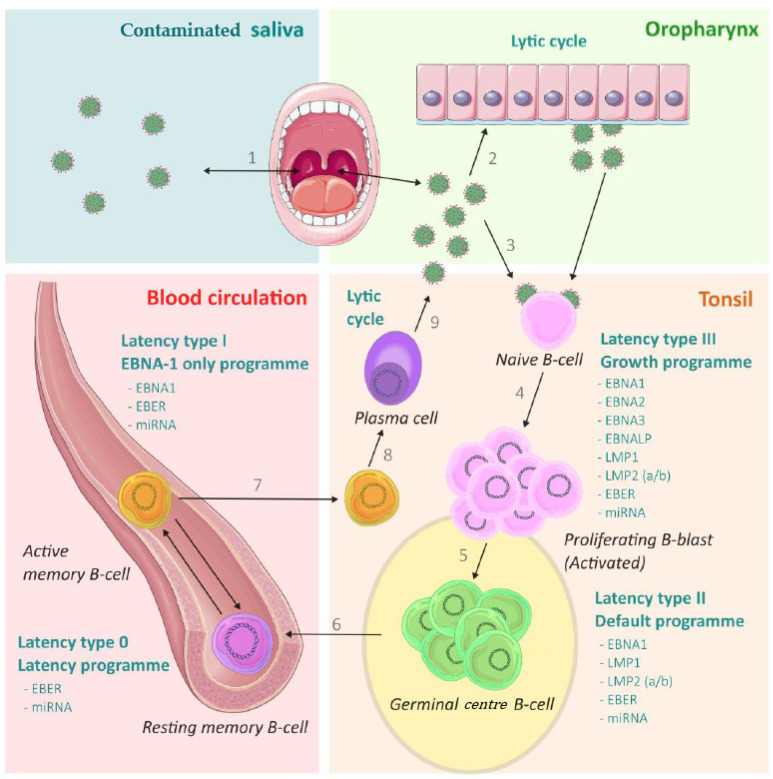
Lifecycle of EBV. After contact with the contaminated saliva (1), EBV infects either the epithelial cells of the oropharynx (2), where it actively replicates, or directly infects naive B-cells (3). The infected naive B-cells go through latency type III and actively proliferate into activated B-blasts or B-cells (4). The activated B-cells migrate to the germinal centres of the tonsils, where latency type II occurs and produces germinal centre B-cells, the lifelong reservoir of EBV (5). The germinal centre B-cells can go into the blood circulation where latency type 0 (resting memory B-cells) or latency type I (active memory B-cells) occur (6). Latency type 0 allows immune escape (since no protein is produced). Latency type I allows the division of B-cells and, thus, the replication of the episomal EBV genome. The active memory B-cells can return to the tonsils (7) and go through a lytic cycle after transformation into plasma cells (8), which produces new virions in the saliva (9). These new virions can therefore infect other naive B-cells (3), epithelial cells of the oropharynx (2) or be excreted in saliva (1).

The cycle of EBV infection includes lytic phases, where the virus actively replicates, and the latent phase, where EBV establishes lifelong persistence (Figure 2). The germinal centre model suggests that EBV persists by mimicking the physiological B-cells maturation process in the germinal centres of lymphoid tissues. This model has been used to describe the pathophysiology of EBV-induced lymphomas [21].

During primary infection after contact with contaminated saliva, the virus can either replicate first in the epithelial cells of the oropharynx or directly infect naive and/or memory B-cells in the lymphoid tissue of the tonsils. These infected naive B-cells will express a set of viral latency genes that will induce B-cells proliferation and prevent their apoptosis. The latent cycle of EBV, which does not produce any virions or lytic cycle proteins, is divided into several steps chronologically described as follows: Type III latency or “growth programme”, during which most of the latent genes are expressed, such as *EBNA1*, *EBNA2*, *EBNA3A/B/C*, *EBNA-LP*, *LMP1* and *LMP2*, as well as many miRNAs. This type III latency allows clonal expansion of infected B-cells [22,23]. Some of these activated B-cells will move in the germinal centre of the tonsils, where type II latency or “default programme” will take place. During this programme, only EBNA1, LMP1 and LMP2, as well as some miRNAs, are produced, transforming infected B-cells in memory B-cells. It is suggested that Hodgkin’s disease originates from these germinal centre B-cells in latency II [21]. Infected memory B-cells will persist lifelong in the germinal centre and serve as a reservoir for EBV. They may reach other secondary lymphoid sites and remain there via the bloodstream. The type I latency or EBNA-1-only programme takes place in the bloodstream. In this programme, only EBNA1 and some miRNAs are produced, allowing these memory B-cells to divide and thus replicate the EBV genome [24]. The germinal centre model predicted that Burkitt’s lymphoma arises from these latency I memory B-cells [21]. Latency 0 also takes place in the bloodstream, where memory B-cells, which do not produce any viral protein, allow EBV to evade the immune system, especially the CD8+ T-cell response [3,25,26,27].

These mature EBV-infected B-cells can switch from the latent stage into lytic replication. Factors inducing this switch are poorly understood [28]. The purpose of the lytic cycle is the production of new infectious virions and is composed of three steps of specific gene expression: immediate-early, early and late. This reactivation is mediated by BZLF-1 and BRLF-1 (immediate-early) and ends with the production of the structural components of EBV, leading to the excretion of EBV virions after the transformation of B-cells into plasma cells. The produced virions can infect oropharyngeal epithelial cells and other B-cells. The infection of oropharyngeal cells results in local replication of the virus and its release into saliva, allowing the infection of new individuals [28,29]. See Figure 2 for an overview of the lifecycle of EBV.

## 3. Latency Proteins

### 3.1. EBNA1

Epstein-Barr nuclear antigen 1 (EBNA1) is a viral protein expressed by EBV that plays a major role in the establishment of EBV latency. It is the only protein expressed during all latent phases in immortalised cells, making it a prime target for the immune system. It is involved in viral DNA replication, expression of other latent genes, maintenance of EBV as episomal DNA, immune evasion of the virus and cell immortalisation. This protein is also involved in the tumorigenesis of some EBV-associated malignancies [30,31].

#### 3.1.1. Higher Anti-EBNA1 IgG Levels in MS Patients Than in Healthy Individuals

Although approximately 95% of the world’s adult population is anti-EBNA1 IgG positive and therefore makes epidemiological studies difficult to carry out, the association between high anti-EBNA1 IgG levels and the occurrence of MS has long been described [32,33,34,35,36,37]. Prospective studies revealed high levels of anti-EBNA1 IgG in the serum of patients before the onset of MS, suggesting that EBV infection precedes MS and that the disease is, therefore, a consequence rather than a cause of EBV infection [38,39,40]. Furthermore, the association between these high levels of anti-EBNA1 IgG and the occurrence of infectious mononucleosis in childhood would further increase the risk of MS [41,42]. A study carried out on MS patients confirms this association between immunity to EBV and MS. The EBV seroconversion does indeed precede the disease, and it increases the risk of MS up to 32-fold [8]. In this study, only one out of 955 MS patients was likely to be not immune to EBV. It has been suggested that high levels of serum anti-EBNA1 IgG may reflect a defective control of EBV infection [43,44].

#### 3.1.2. Modification of Anti-EBNA1 IgG Levels to Predict Outcome in MS Patients

Given the strong correlation between the onset of MS and high levels of anti-EBNA1 IgG, many studies have investigated whether it is possible to predict the course of the disease in MS patients. Some studies demonstrated a correlation between high anti-EBNA1 IgG levels and an increased MRI disease activity, as well as an association between high anti-EBNA1 IgG levels and impairment of the Expanded Disability Status Scale (EDSS), which is used to assess neurological dysfunction in MS patients [45,46,47].

In a follow-up study involving 69 patients, high anti-EBNA1 IgG titers are reported during the inflammatory phases of the disease, and these high titers indicate a longer active phase in patients with RRMS. Authors suggest that these high titers are also associated with the severity of relapses as well as the location of lesions. Anti-EBNA1 IgG titers could therefore serve as a useful biomarker for monitoring and prognosis of MS patients [48].

However, conflicting studies have not found an association between elevated plasma anti-EBNA1 IgG and the occurrence of radiological lesions and/or altered EDSS scale [49]. Anti-EBNA1 IgG levels also do not seem to correlate with the presence and/or the intensity of fatigue in MS patients, which is one of the most frequent symptoms [50]. Other studies have also shown that high levels of anti-EBNA1 IgG do not specifically correlate with the risk of progression from CIS to MS [50] or with MS severity [51,52]. Furthermore, anti-EBNA1 IgG levels do not appear to decrease after treatment with natalizumab or IFN-β, although these treatments are associated with clinical improvement of the disease [53], suggesting that anti-EBNA1 IgG level is not a good marker for monitoring MS patients under treatment. It is, therefore, difficult at this time to establish a clear correlation between anti-EBNA1 IgG levels and the prognosis of MS patients due to the contradictory results in the different studies.

#### 3.1.3. Changes in Anti-EBNA1 IgG Levels Associated with Other MS Risk Factors

Hypovitaminosis D is considered a major risk factor in MS, although it is not yet clear whether there may be a direct effect of this deficiency or whether it simply reflects the geographical disparity of MS, characterised by a high prevalence in some countries less exposed to sunlight [54,55]. An inverse relationship between anti-EBNA1 IgG levels and 25-hydroxyvitamin D (25-OH Vitamin D) levels is demonstrated in some studies [56,57]. In the study of Wergeland et al., this relationship was observed only in RRMS patients with the HLA-DRB1 15*01 haplotype, suggesting the involvement of genetic and environmental factors in the modulation of anti-EBNA1 IgG levels [57]. This genetic susceptibility has also been reported in other studies [58,59]. Several studies have also investigated the potential beneficial effect of vitamin D supplementation in RRMS patients on a decrease in anti-EBNA1 IgG levels [60,61,62]. Interestingly, supplementing RRMS patients with vitamin D seems to decrease anti-EBNA1 IgG titers. The study of Røsjø et al. showed a decrease in anti-EBNA1 IgG levels between day 0 and week 48, but not between day 0 and week 96, in RRMS patients supplemented with high-dose vitamin D3. This association, although quite weak and requiring further investigations, may suggest a beneficial role for high-dose vitamin D3 supplementation in the immune response to EBV [60]. Another epidemiological study also found a correlation between low sunlight exposure and high levels of anti-EBNA1 IgG in MS patients. However, it is not clear whether this is directly due to the low sunlight exposure or the consequent lack of vitamin D [55].

Another study did not find a significant correlation between vitamin D levels and anti-EBNA1 IgG levels. These discrepant results could potentially be explained by selection bias or by the genetics of the studied population [63,64].

Interestingly, another study found a correlation between levels of multiple sclerosis-associated retroviruses (MSRV) DNA in peripheral mononuclear cells and serum vitamin D concentration in RRMS patients, suggesting a potential role for vitamin D and human endogenous retrovirus (HERV) in MS [65].

#### 3.1.4. Higher Anti-EBNA1 IgG Correlates with Presence of Oligoclonal Bands in the CNS

Another important aspect regarding EBNA1 is that high serum anti-EBNA1 IgG levels also correlate with the presence of oligoclonal bands (OCBs) in the cerebrospinal fluid (CSF) of MS patients [66,67]. These OCBs or oligoclonal immunoglobulins are a major hallmark of MS [68], suggesting a link between EBV immunoreactivity and MS. Many studies attempted to characterise these OCBs. However, the targets of these antibodies are still poorly understood [69]. Since OCBs are the result of intrathecal production of IgG [70], and MS patients have higher levels of anti-EBNA1 IgG in both blood and CSF than non-MS patients, it was speculated that these OCBs might reflect increased production of anti-EBNA1 IgG locally in the CNS. However, this could not be demonstrated by comparing the serum/intrathecal IgG ratio of MS patients to a control population [71]. A slight increase in local anti-EBNA1 IgG production has been shown in a very small subgroup of RRMS patients, which is not representative of the whole group of RRMS patients [47,72].

Two more recent studies investigated the antigen specificity of IgG antibodies from the CSF of MS patients [69,73]. One team demonstrated that some targeted peptides showed sequence homologies with EBNA1 and EBNA2 [69]. However, another interesting study has shown that these antibodies recognise mainly self-proteins derived from the cellular destruction of CNS components [74].

In conclusion, although there is a significant association between serum anti-EBNA1 IgG titers and the presence of OCBs in the CSF of MS patients [66,67], it seems less likely that these bands are only the result of local IgG production against EBV. However, it has been suggested that those antibodies may cross-react between the EBV component and some CNS particles.

#### 3.1.5. Cross-Reaction

It has been shown that the antibodies produced by a B cell-derived clone, isolated from the CNS of MS patients, can cross-react with a high affinity for both the AA386-405 epitope of EBNA1 and for GlialCAM [75], a glial cell adhesion protein involved in many neurological diseases [76]. In mice, immunisation with EBNA1 AA386-405 induces a strong response against GlialCAM and worsens the previously induced experimental autoimmune encephalopathy [75,77]. However, this cross-reaction was only found in 20–25% of MS patients [75].

The hypothesis of cross-reaction is also evoked for several other proteins: studies have shown higher levels of autoantibodies to chloride-channel protein anoctamin 2 (ANO2) in MS patients and the possible cross-reaction between these autoantibodies and EBNA1 protein. The presence of autoantibodies against ANO2 was also associated with HLA-DRB1*15, which is a major risk factor for the development of MS [78,79]. Anti-EBNA1 antibodies are also suspected to react with alpha-crystallin B chain or CRYAB, a protein with sequence homologies to EBNA1 [80]. It is also important to note that antibodies targeting other EBV proteins, such as BFRF3 and BRRF2, can also react to self-proteins [81]. Finally, the possibility of cross-reaction between antibodies to EBNA1 and self-components is also described in other autoimmune diseases such as lupus erythematosus (SLE) [82,83,84].

### 3.2. EBNA2

Epstein-Barr nuclear antigen 2 or EBNA2 is one of the EBV proteins expressed during the latency period in infected B-cells. This protein plays a key role in the growth programme (latency III) of these B-cells. It is a transactivator able to up-regulate the expression of certain viral genes (*EBNA1-6*, *LMP1*, *LMP2a/b*) and interact with numerous cellular genes of the infected host [85,86]. It has been shown that EBNA2 is able to induce or repress many cellular genes involved in cell cycle regulation, cell signalling or chemokines expression [87].

A study demonstrated that high levels of anti-EBNA2 antibodies are correlated with the risk of MS. These antibodies appear before the onset of MS, as in the case of anti-EBNA1 antibodies. A 4-fold increase in anti-EBNA2 levels leads to a 4-fold increase in the risk of developing MS [88].

Interestingly, one team demonstrated that some B-cells in the brain of MS patients express EBNA2 and LMP1 simultaneously in the perivascular space of white matter [26]. The expression of these two proteins is the hallmark of latency III. However, other studies have also demonstrated the presence of other EBV-specific proteins (lytic and latent) in the CNS of MS patients.

### 3.3. LMP1, LMP2a and LMP2b

Latent membrane proteins (LMP) 1, 2a and 2b are EBV latent proteins expressed in naive B-cells during the EBV growth programme or latency III in concert with other latent proteins and in the germinal centre memory B-cells during the default programme or latency II. The *LMP2a* transcript is sometimes found in infected circulating memory B-cells or latency I [26,27].

LMP1 and LMP2A are known to mimic the signal presented to B-cells via CD40 (for LMP1) and via the B-cell receptor (for LMP2A), allowing B-cells to survive and grow independently of the required interaction with CD4+ T cells [27,89]. This shows how important these proteins are in viral latency.

#### 3.3.1. Presence of LMP1 and LMP2 in the CNS of MS Patients

EBV proteins LMP1 and LMP2 were identified in several studies which tried to find out whether or not MS patients have particular inflammatory features in the CNS and whether this inflammation is mediated by EBV latency.

B-cells infiltrating the brain in MS patients have been shown to strongly express LMP2-A, in addition to expressing BAFF (B-cell activating factor), a cytokine involved in B-cell activation. Increased BAFF production is found in many other autoimmune diseases involving B-cells [89]. This study also demonstrated the presence of LMP2-A in white matter lesions, perivascular cuffs and lymphocyte aggregates in the brain of MS patients, along with LMP1 and EBER. This profile, in the absence of EBNA2, suggests that a latency stage II or “default programme” takes place in the CNS [89]. This default programme transforms infected B-cells into memory B-cells in germinal centres, making these memory B-cells a reservoir for EBV latency. Other studies attempting to identify EBV proteins by immunohistochemistry (IHC) and in situ hybridization (ISH) on samples from CNS lesions of MS patients have demonstrated the presence of LMP1, with higher levels in MS patients than in a control group [90]. Other proteins from all stages of viral replication (lytic and latent) were found in addition to LMPs in such studies, indicating a broad protein expression of EBV in the CNS of MS patients. These findings raise interrogations about the ability of EBV to alternate between lytic and latent phases in the CNS of MS patients and if these cycles are disrupted [91].

In contrast, some studies have failed to isolate evidence of EBV in the CNS of MS patients [92,93].

Interestingly, Serafini et al. found that CD8+ T cells infiltrating the CNS of MS patients recognise the majority of EBV latent proteins (including LMP1 and LMP2A) as well as many lytic cycle proteins, corroborating their previous findings. This finding is consistent with the idea of a local EBV infection in the CNS with a T cell response against EBV, probably linked to the pathophysiology of MS [94].

#### 3.3.2. Presence of LMP1 and LMP2a in Exosomes Derived from EBV-Infected Cells

Exosomes are small vesicles containing any kind of protein, lipid and genetic content (such as non-coding miRNAs) which can remotely modulate cellular activity and microenvironment and even support inflammation or immune escape. It was shown that exosomes from EBV-infected B-cells contain viral latency products such as LMP1 and LMP2, which could lead to the delivery of EBV products into non-infected cells. Blood monocyte-derived macrophages of RRMS patients in the active inflammatory phase can produce exosomes harbouring LMP1 and LMP2A at higher levels than a control group of healthy individuals and a control group of stable MS patients, suggesting a correlation between these EBV protein-containing exosomes and disease severity [95].

#### 3.3.3. Variations and Cross-Reaction

Of note, LMP1 (but also EBNA1) variants do not seem to be associated with an increased risk of MS or increased anti-EBNA IgG levels [96]. Another interesting finding suggests that some antibodies in MS patients can cross-react with myelin basic protein (MBP) and LMP1 in vitro [97].

## 4. Lytic Cycle Proteins

The genes encoding the EBV lytic cycle proteins are essential for the replication of the virus and, therefore, its dissemination. Many of these genes encoding lytic cycle proteins are still unknown or poorly identified. Their role is also widely suspected in EBV oncogenesis. These lytic cycle proteins are divided into three phases: Immediate-early (expression of the two viral transactivators BZLF-1 and BRLF-1 inducing entry into lytic phase), early and late (coding for structural viral proteins) [98].

### 4.1. Presence of Lytic Cycle Proteins in the CNS of MS Patients

Many studies using IHC, ISH or real-time polymerase chain reaction (RT-PCR) on samples from the CNS of dead MS patients at different stages of the disease revealed the presence of immediate-early proteins and/or immediate-early genes, suggesting the presence of a lytic cycle in the CNS of MS patients in addition to the presence of B-cells in latent phase as seen previously with LMP1 and LMP2A.

One team was able to detect BZLF-1 in the inflamed meninges and in the periphery of follicle-like structures (FLS). These structures are aggregates of B-cells located in the subarachnoid space, especially in the cortical folds, sharing some characteristics with germinal centres (germinal centres are important actors in the persistence of EBV—Figure 2). They also observed the presence of BZLF-1 in perivascular cuffs of inflamed vessels and in active white matter lesions but not in demyelinated, chronic active and inactive white matter lesions, suggesting that reactivations occur in acute inflammation. Moreover, many of the cells infiltrating the meninges and active lesions were plasmablasts or plasma cells. The transformation of memory B-cells into these plasma cells is an important step in the lytic reactivation of EBV. Another study also demonstrated the presence of B-cells and plasma cells expressing EBV immediate-early proteins in the perivascular cuffs of MS patients [99]. Altogether, these observations suggest that lytic EBV reactivations occur in acute CNS inflammation in MS patients [100].

B-cells and plasma cells expressing BFRF-1, an early protein, are also present in FLS and in acute brain lesions of MS patients. These B-cells were not detectable in chronic active lesions, also linking reactivations to acute inflammation [26,101].

Two case reports investigating brain lesions of patients who died after severe MS attacks (induced by natalizumab discontinuation) further support the role of EBV reactivation in the acute phases of the disease. They found a significantly higher frequency of BZLF-1 and BFRF-1 in the brain of these patients than in their previous studies of chronic MS subjects. These findings suggest that EBV reactivation in the white matter is associated with significant inflammatory tissue destruction and neurological deterioration [102,103].

Other studies have also found a higher frequency of BZLF-1 in the brain of MS patients than in the brain of healthy controls. However, the high frequency of BZLF-1 was shown in chronic lesions but not in active chronic lesions [90]. Hassani et al. found cells expressing *BZLF-1* in the brain of MS patients, but scattered in the whole brain and not localised to specific structures [104], while in other studies, EBV markers (lytic or latent) were not found in the brain of MS patients at all [105,106,107].

All these differences in the presence but also in the localisation of these lytic cycle proteins or genes can be explained by several factors: potential biases in the selection of the population, the stage of the disease, the sampling of representative areas such as FLS or active MS lesions, the samples preparation, as well as the choice of detection technique, such as IHC, ISH or RT-PCR. All these parameters make the standardisation of such studies more complex [108,109].

### 4.2. Weakened T-Cell Response against Lytic Cycle Proteins in MS Patients

The CD8+ T cell response to these lytic cycle proteins has been investigated. Indeed, a peripheral increase in the number of specific CD8+ T cells targeting lytic cycle proteins is described [110]. These findings are in agreement with the results of other teams, describing an increase in the number of peripheral CD8+ T cells reacting against EBV lytic cycle proteins (BZLF-1 and BMFL-1) of MS patients with active lesions on MRI, suggesting a correlation between the elevation of these lytic cycle protein-specific CD8+ T cells and active disease phases [100]. However, the lack of plasma viral load elevation during these active phases compared to healthy subjects suggests that the fluctuation in the number of specific CD8+ T cells in MS patients is not the result of a systemic control deficiency but rather a local control failure in an immune privileged site such as the CNS [100]. Another study corroborates this particular CD8+ T-cell activity directed mainly against lytic cycle proteins (immediate early, early and late) intrathecally. They could not find intrathecal T-cell activity directed against other EBV proteins, such as EBNA1 [111].

The Pender et al. team reported a decrease in the activity (cytokine secretion) of the CD8+ T-cells specific to EBV lytic cycle proteins (in the periphery) at the beginning of MS and throughout the course of the disease. The number of CD8+ T-cells directed against latent proteins was higher but with reduced polyfunctionality, suggesting an “exhaustion” of these T cells. This constatation was not observed for CMV, suggesting that it is indeed a defective control specific to EBV, particularly in the lytic phase. It was also suggested that during attacks, EBV-specific CD4+ and CD8+ T-cell populations increase, and this rate progressively decreases with the evolution of the disease, arguing for the exhaustion of lymphocytes. It is not clear why this diminished CD8+ T-cell response affects the lytic antigens response specifically, but it shows the importance of the control of lytic phases in the development of MS. Another interesting finding is that anti-EBNA1 IgG levels are inversely correlated with the number of lytic antigen-specific CD8+ T cells, also suggesting that defective control of the lytic phase by these cells would result in EBV escaping from the immune system, inducing a stronger humoral response against this virus. They hypothesised that this defective control would lead to an accumulation of EBV-positive autoreactive B-cells in the CNS of MS patients [44].

## 5. Epstein-Barr Virus miRNAs

EBV miRNAs are non-coding RNAs that regulate post-transcriptional gene expression and are expressed very abundantly in EBV latently infected cells [112,113]. Widely studied in the context of EBV-associated tumours, more than 40 miRNAs essential for the EBV lifecycle are encoded by the virus and are grouped in two clusters located into BART transcript and around the BHFR1 gene (Figure 1) [114]. These miRNAs interfere with many functions, such as cell apoptosis, antigen presentation and recognition, and B-cell transformation. The disruption of these functions, therefore, contributes to the escape of the virus and infected cells from the immune system [113,115]. EBV miRNAs appear to be expressed in both latent and lytic phases, and their role in the transition from one phase to the other is strongly suspected [114,116,117,118,119,120,121].

EBV miRNAs have been identified in numerous studies using ISH on CNS samples from MS patients [99,108,120,121], although this has also been demonstrated at lower rates in non-MS patients [104].

Exosomes also contain many EBV miRNAs [95,113]. The presence of these EBV miRNAs in the CNS of MS patients should therefore be investigated in order to determine whether they are indeed produced “locally” or whether remote production may be found in the CNS via these exosomes [116].

Table 1 summarizes the findings for markers of EBV infection in patients with MS.

## 6. Conclusions

Markers of EBV infection can be found in MS patients, which argues in favour of a relationship between EBV and MS. How EBV may play a role in the pathogenesis of MS remains to be discovered. Nevertheless, observations regarding the association between this virus and the disease open the prospect of designing new treatments for MS by combating the virus.

## Figures and Tables

**Table 1:** Markers of EBV infection in patients with multiple sclerosis.

Marker	Expression	Observations in Patients with Multiple Sclerosis
EBNA1	Latency III Latency II Latency I	- High anti-EBNA1 IgG levels in MS patients - Suspected correlation between anti-EBNA1 IgG levels and clinical outcomes of MS patients - Anti-EBNA1 IgG levels inversely correlated with vitamin D levels in the serum of MS patients - Anti-EBNA1 IgG levels correlated with the presence of OCBs in the CSF of MS patients - Cross-reaction between anti-EBNA1 IgG and self-proteins (GlialCAM, CRYAB, ANO2)
EBNA2	Latency III	- High anti-EBNA2 IgG levels correlated with an increased risk of MS - Detection of EBNA2 in the CNS of MS patients
LMP1, LMP2a LMP2b	Latency III Latency II	- Detection of LMP1 and LMP2a in the CNS of MS patients - Higher LMP1 and LMP2a frequency in EBV infected B-cells derived exosomes in MS patients than in control groups - Cross-reaction between LMP1 and myelin basic protein (MBP) in vitro
Lytic cycle proteins	Lytic cycle	- Detection of immediate-early lytic cycle proteins in the CNS of MS patients - Weakened T-cell response against lytic cycle proteins in MS patients
miRNA	Latency III Latency II Latency I Latency 0 Lytic cycle	- Presence of EBV miRNAs in the CNS of MS patients

## Data Availability

Not applicable.
